# Study on the Regulatory Mechanism of the Lipid Metabolism Pathways during Chicken Male Germ Cell Differentiation Based on RNA-Seq

**DOI:** 10.1371/journal.pone.0109469

**Published:** 2015-02-06

**Authors:** Qisheng Zuo, Dong Li, Lei Zhang, Ahmed Kamel Elsayed, Chao Lian, Qingqing Shi, Zhentao Zhang, Rui Zhu, Yinjie Wang, Kai Jin, Yani Zhang, Bichun Li

**Affiliations:** 1 Key Laboratory of Animal Breeding Reproduction and Molecular Design for Jiangsu Province, College of Animal Science and Technology, Yangzhou University, Yangzhou, 225009, China; 2 College of Veterinary medicine, Suez Canal University, Ismailia, 41522, Egypt; Northeast Ohio Medical University, UNITED STATES

## Abstract

Here, we explore the regulatory mechanism of lipid metabolic signaling pathways and related genes during differentiation of male germ cells in chickens, with the hope that better understanding of these pathways may improve in vitro induction. Fluorescence-activated cell sorting was used to obtain highly purified cultures of embryonic stem cells (ESCs), primitive germ cells (PGCs), and spermatogonial stem cells (SSCs). The total RNA was then extracted from each type of cell. High-throughput analysis methods (RNA-seq) were used to sequence the transcriptome of these cells. Gene Ontology (GO) analysis and the KEGG database were used to identify lipid metabolism pathways and related genes. Retinoic acid (RA), the end-product of the retinol metabolism pathway, induced in vitro differentiation of ESC into male germ cells. Quantitative real-time PCR (qRT-PCR) was used to detect changes in the expression of the genes involved in the retinol metabolic pathways. From the results of RNA-seq and the database analyses, we concluded that there are 328 genes in 27 lipid metabolic pathways continuously involved in lipid metabolism during the differentiation of ESC into SSC in vivo, including retinol metabolism. Alcohol dehydrogenase 5 (ADH5) and aldehyde dehydrogenase 1 family member A1 (ALDH1A1) are involved in RA synthesis in the cell. ADH5 was specifically expressed in PGC in our experiments and aldehyde dehydrogenase 1 family member A1 (ALDH1A1) persistently increased throughout development. CYP26b1, a member of the cytochrome P450 superfamily, is involved in the degradation of RA. Expression of CYP26b1, in contrast, decreased throughout development. Exogenous RA in the culture medium induced differentiation of ESC to SSC-like cells. The expression patterns of ADH5, ALDH1A1, and CYP26b1 were consistent with RNA-seq results. We conclude that the retinol metabolism pathway plays an important role in the process of chicken male germ cell differentiation.

## Introduction

Germ cell differentiation is complex, involving regulation of many genes and cellular regulation pathways. Processes such as the differentiation of telogonia into sperm or oocytes have been the focus of intense study, with the hope that understanding them will shed light on the regulatory mechanism of germ cell development and differentiation and may be applicable to the problem of infertility.

A number of studies highlight the link between metabolism and cell growth and differentiation, particularly the role of lipid metabolism. In a study of the regulation of lipid metabolism during rat adipoctye differentiation, Lu et al. [1] found that the expression of the sterol regulatory element binding protein SREBP-1c was associated with early differentiation of fat cells. Kee et al. [2] successfully induced mice embryonic stem cells to differentiate into sperm and oocytes by manipulating chemicals and vitamins. When Aflatoonian and Moore [3] stimulated in vitro human embryonic stem cells to differentiate into sperm, double hydrogen testosterone (DHT) was released into the external medium and Hiibner et al. [4] detected estradiol (E2) in the course of inducing mouse embryonic stem cells to differentiate into spermatagonial stem cells. These studies strongly suggest that lipid metabolism is important in cell differentiation.

There have been many studies on the regulation of lipid metabolism in the in vitro induction of chicken male germ cell differentiation, but these have not yielded a complete understanding of the process and the selection of the inducer remains relatively blind. Here, we attempt to identify the genes related to lipid metabolism during male germ cell generation in chickens as well as their regulation and signaling pathways.

Analyzing and characterizing the transcriptome is central to understanding the function of the genes in any particular cell. RNA sequencing (RNA-seq) technology [5–10], therefore, was central to this study, and we have taken advantage of RNA-seq technology. We have standardized and improved the in vitro culture system for the generation of male germ cells.

## Materials and Methods

### Experimental specimens

Procedures involving animals and their care conformed to the U.S. National Institute of Health guidelines (NIH Pub. No. 85–23, revised 1996) and were approved by the laboratory-animal management and experimental-animal ethics committee of Yanzhou University.

Eggs were collected shortly after fertilization from the poultry institute of the Chinese Academy of Agricultural Sciences Experimental Poultry Farm. A total of 18,340 eggs were used in this experiment, which were divided into three groups as three biological replicates. The eggs used for collection of embryonic stem cells (ESC) were used immediately, while those used for isolation of primordial germ cell (PGC) or isolation of spermatogonial stem cell (SSC) were incubated at 37°C and 75% relative humidity for 5.5 and 19 days, respectively [11].

Dulbecco’s modified eagle medium (DMEM) and fetal bovine serum (FBS) were supplied by Gibco (USA) and mitomycin-C was supplied by Roche. β-mercaptoethanol, chicken serum, L-glutamine, sodium pyruvate, trypsin, collagen enzyme I, human stem cell factor (hSCF), basic fibroblast growth factor (bFGF), human insulin-like growth factor (hIGF), and murine leukemia inhibitory factor (mUF) were acquired from Sigma-Aldrich. The antibodies used were: antibody to SSEA-1 (Biolegend, San Diego, CA, USA; dilution ratio 1:100), antibody to Sox2 (abcam, Cambridge, England; dilution ratio 1:100), antibody to C-kit (SouthernBiotech, Birmingham, AL, USA; dilution ratio 1:100), antibodies to α-6 and β-1 integrins (Millipore; dilution ratio 1:100), and goat anti-mouse IgM (flourescein isothyocyanate [FITC] labeled; Bio-Synthesis, Inc., Texas, USA; dilution ratio, 1:100).

### Isolation and culture of ESC, PGC, and SSC

Separation and cultivation of ESC, PGC, and SSC was performed as has been described [12–14].

### Sex determination

Genomic DNA from ESCs and PGCs was extracted to identify male and female germ cells through PCR amplification using the primer sequences: F: GTTACTGATTCGTCTACGAGA and R: ATTGAAATGATCCAGTGCTTG in a polymerase chain reaction (PCR) system consisting of thirty cycles of: 98°C for 10 s, 49°C for 5 s, 72°C, for 30 s followed by long-term storage at 4°C. The male germ cells were used in RNA-seq analysis.

### Flow cytometry cell sorting

Highly purified cells were required for the next phase of the experiment. These were acquired by using two antibodies in combination to label and select cell types. Antibodies to SSEA-1 and Sox2 were used to mark ESCs, antibodies to C-kit and SSEA-1 were used to mark PGCs, and antibodies to the α-6 and β-1 integrins were used to mark SSCs.

### RNA sequencing

In this endeavor, we were guided by the mRNA-seq procedures of Illumina, Inc., (USA). Fifty-nanogram cell tissue samples were sequenced using the HiSeq 2000 system (Illumina, Inc., USA) by Shanghai OE Biotech. Co., Ltd. The results were compared with the database and annotations of every gene for subsequent experimental analysis.

### In vitro induction experiment

We used retinoic acid (RA) to induce the differentiation of chicken ESCs into male germ cells. Third-generation ESCs were cultured onto a 24-well plate with a feeder layer with a density of 10^5^ cells per well in culture medium containing 10 μM RA [15]. The cells were collected every two days after induction. Quantitative real-time PCR (qRT-PCR) was used to expression of the marker genes by ESC, PGC, and SSC cultures, as well as the expression of key genes of retinol metabolism.

### Quantitative Real Time PCR

Total RNA was extracted with the RNeasy kit (Qiagen). Reverse transcription was performed to create cDNA to serve as a template for qRT-PCR. qRT-PCR was performed according to the instructions provided in the fluorescence quantitative PCR kit, using SYBR as the fluorescent reagent and 7500 System fluorescence quantitative instrument (Applied Biosystems). The data were analyzed with the 2-^Δ Δ^ Ct relative quantitative method in Microsoft Excel software. The primer sequences were: for CYP26b1 (Gene bank accession: XM_426366.2), F: ACATAACCCGAAAGCAGTGG, R: TCATCTCTGTGCCCTACACG; for ALDH1A1 (Gene bank accession: NM_204577.3), F: CAGCAGGGAAGACCAATCTG, R:CGGCGAACAAACTCAT CATA; For ADH5 (Gene bank accession:NM_001031152.1), F: GAGTTGGGCTG GCTACTGTT, R:CCTCCTGTATGGGCTTCTCA.

### Data analysis

The differentially expressed genes between different samples were compared by the method of PRKM. The false discovery rate (FDR) was set at 0.0001 to determine the threshold of the P-value in multiple tests. The absolute value of |log 2Ratio|≥1 was used as the threshold for significance of the gene expression difference between the transcriptome and database. DAVID (http://david.abcc.ncifcrf.gov/home.jsp), FunNet (http://www.funnet.info/) and WEGO (http://wego.genomics.org.cn/cgi-bin/wego/index.pl) were used to screen the key genes and signaling pathways.

## Results

### Separation of Male ESCs, PGCs. and SSCs

**Figure 1:**
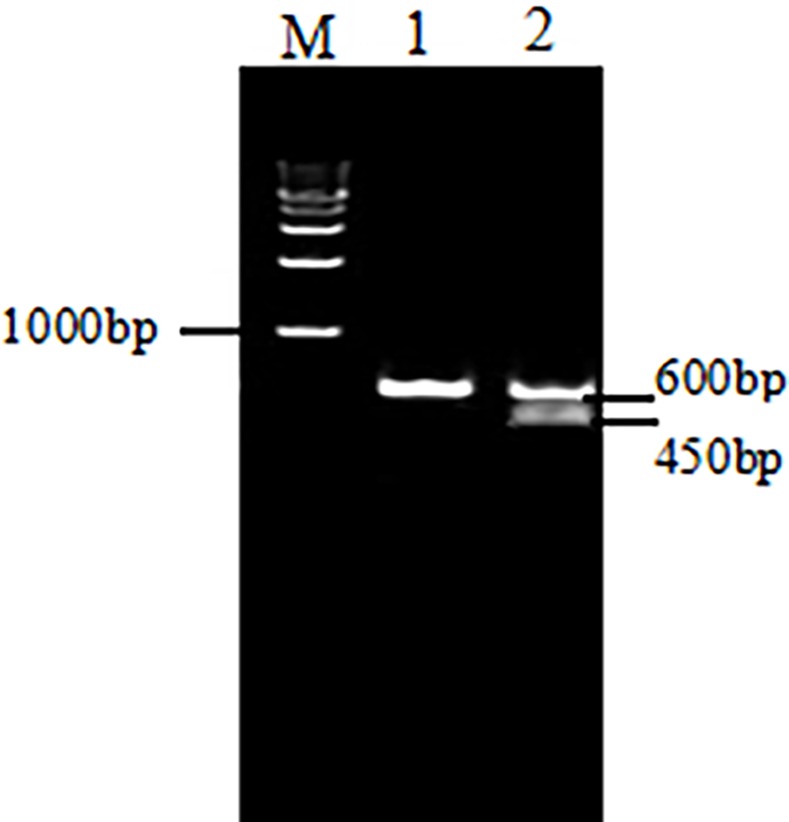
Sex determination of the isolated cells. The sex of the isolated cells was determined through PCR amplification of the *CDH-W* gene. In this process, males could be detected by the single fragment at 600 bp (Line 1) and the female by the presence of two fragments, at 600 bp and 450 bp (Line 2). (M): 1000 bp Marker.

**Figure 2:**
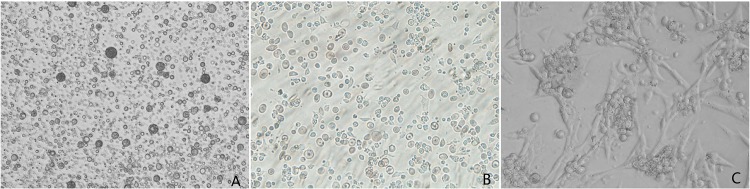
Morphological characters of chicken ESCs, PGCs, and SSCs. *A*: The clones of ESCs resemble a bird’s nest with clear edges. *B*: PGCs are larger than ESCs with more obvious nuclei and clearly visible areas around the cells. *C*: The SSCs are large and clump into a mass that resembles a bunch of grapes. Magnification: 400×.

In this study, we needed cultures composed of cells that were all-male and of the same cell type. Sex discrimination in chickens can be achieved by using *CHD-W*, a gene on the W chromosome that is present only in females, as a marker for sex. We used specific primers in PCR to amplify *CHD-W*. The results are shown in Fig. 1. Females, of genotype ZW, had two DNA fragments, at 600 bp and 450 bp. Males, of genotype ZZ, had only one fragment at 600 bp. Once pure male cells had been acquired, flow cytometry sorting was used to separate the three cell types, employing labeled antibodies specific for cell type. ESCs, PGCs, and SSCs are morphologically distinct and their cultures can be easily distinguished (Fig. 2).

### Analysis of expression differences of lipid metabolism genes

We next wished to examine the genes regulating chicken male germ cell development and differentiation. RNA extracted from the three purified cultures of male germ cells was sequenced. Then, we compared the transcriptomes with the available databases. The false discovery rate (FDR) was set at 0.0001 to determine the threshold of the P-value in multiple tests. The absolute value of |log 2Ratio|≥1 was used as the threshold for significance of the gene expression difference between the transcriptome and database.

**Figure 3:**
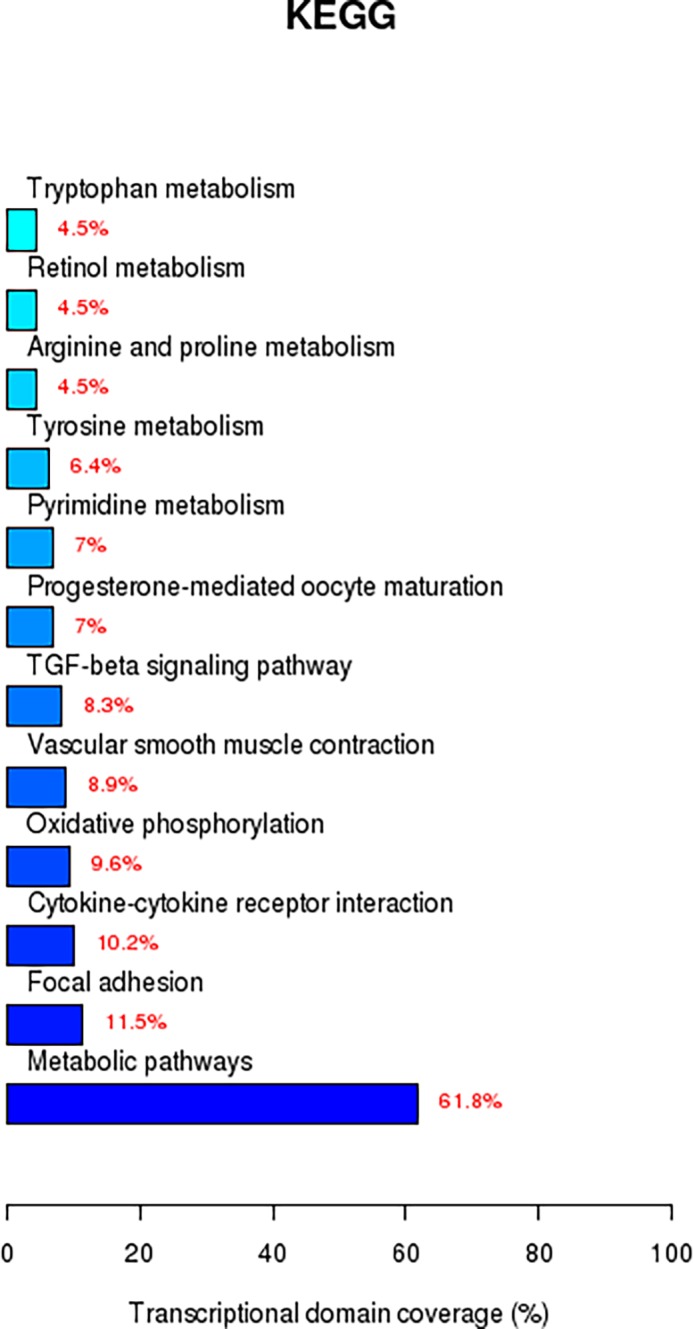
Enrichment in the transcriptomes of genes involved in lipid metabolism as revealed by the FUNNET database. KEGG database resulted in 328 different genes enriched in 27 pathways related to lipid metabolism. Of these genes, 4.5% were related to retinol metabolism.

Gene ontology (GO) analysis was used for detection of differences in gene function, annotation, and classification between GO categories and the transcriptome. From this analysis, we identified 697 genes enriched in the transcriptome that participated in the metabolic regulation of biological macromolecules, including lipid, protein, and glycol metabolism. GO analysis, combined with cellular pathway analysis through FUNNET and DAVID databases, also showed enrichment of amino acid metabolism pathways, lipid metabolism pathways, and other pathways. The KEGG database revealed 328 genes that were enriched in the transcriptome that participated in 27 pathways related to lipid metabolism. The differences were particularly notable in steroid biosynthesis, primary bile acid synthesis, retinol metabolism, fatty acid metabolism, and triglyceride metabolism. (Fig. 3, Table 1).

**Table 1:** Differences in gene enrichment of lipid metabolic pathways.

GENE NAME	GENE ID	ESC EXPRESSION	PGC EXPRESSION	SSC EXPRESSION
CYP26C1	XM_421678.2	42	73	8
LOC769504	XM_001231508.1	0	28	3
CYP26A1	NM_001001129.1	77	405	7
CYP26B1	XM_426366.2	0	542	1652
CYP2C18	NM_001001757.1	10	130	13
CYP2H1	NM_001001616.1	14	542	59
CYP3A37	NM_001001751.1	10	130	13
CYP3A80	XM_414782.2	20	444	201
ALDH1A2	NM_204995.1	627	11929	8356
ALDH8A1	XM_419732.1	0	44	2
ALDH1A1	NM_204577.3	50	3619	40038
ADH	XM_001234262.1	92	1719	57
ADH1B	NM_205092.1	40	408	17
ADH5	NM_001031152.1	5905	14789	5941
CYP1A1	NM_205146.1	5	2809	62
CYP1A4	NM_205147.1	9	1718	387

### Analysis of lipid metabolism and the retinol metabolic pathway

The KEGG database analysis reveals that genes involved in lipid metabolism were enriched in the transcriptomes of these cells. There is also evidence that RA regulates lipid metabolism and can induce differentiation of germ cells, so we studied expression of genes involved in the retinol biosynthetic pathway in the three cell types.

**Figure 4:**
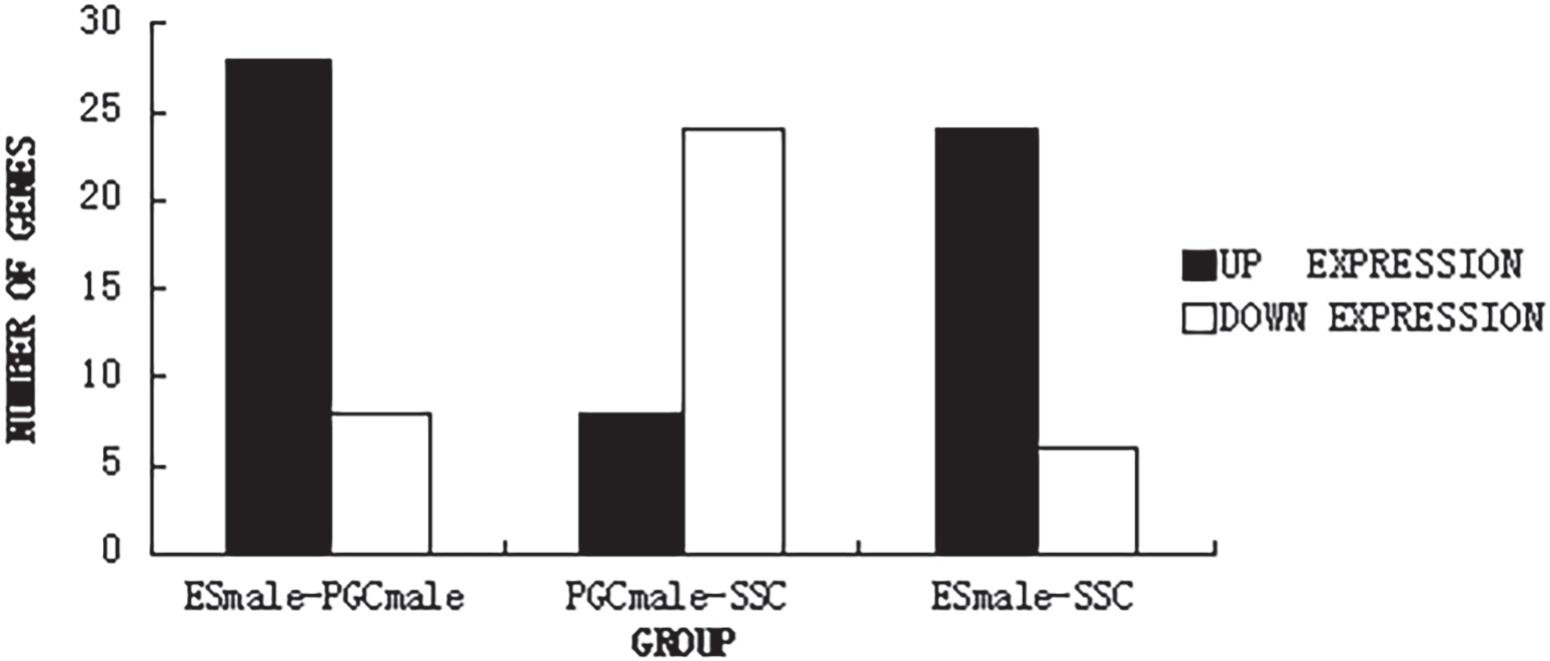
The distribution and expression of genes related to retinol metabolism pathway during germ cell differentiation. There are 36 genes involved in differentiation of ESCs to PGCs. Twenty-eight were up-regulated and eight were down-regulated during the process. In the differentiation of PGCs to SSCs, 32 genes were involved; 28 were up-regulated and eight were down-regulated.

Of the 328 genes related to lipid metabolism that were enriched in the transcriptomes, 48 participated in retinol metabolism. These 48 genes had different patterns of expression in ESCs, PGCs, and SSCs (Fig. 4).

We used Wayne analysis of ES to analyze the expression of the genes in the retinol biosynthesis pathway of the cell types. In PGCs and SSCs, there were 23 genes related to regulation of retinol biosynthesis that were persistently expressed. During the process of differentiation of ESCs to SSCs, eighteen of these genes were initially highly expressed followed by decline in expression, expression of two steadily increased, two were highly expressed after a time lag, while expression of one gene steadily declined during the differentiation process.

**Figure 5:**
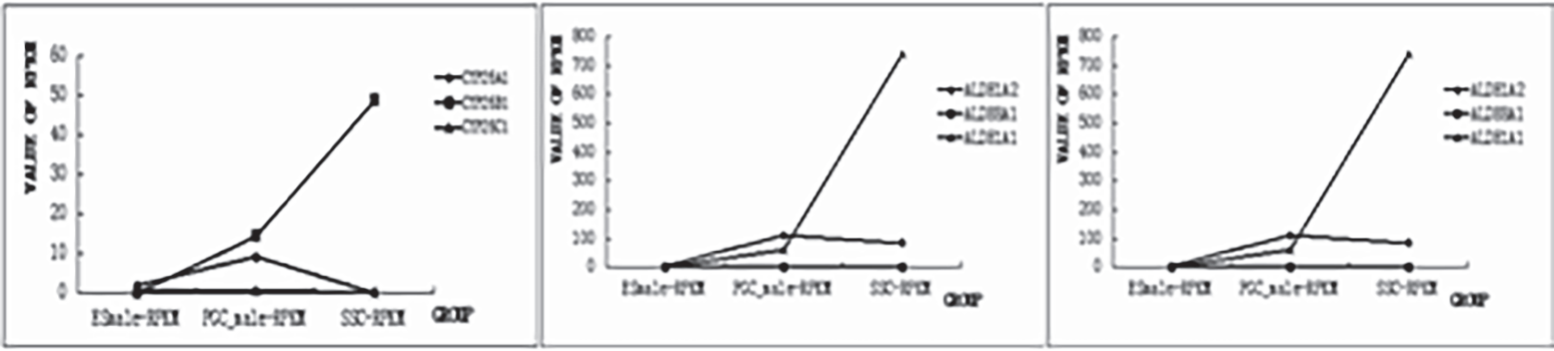
Expression of the gene families CYP26, ALDH, and ADH during differentiation. RNA-seq results showed high expression of CYP26, ALDH, and ADH families, suggesting important roles for these enzymes in germ cell differentiation, particularly CYP26b1, ALDH1A1, and ADH5 genes, which are the members of these families that are present in chickens.

According to the literature, summarized in Table 2 and Fig. 5, acetaldehyde dehydrogenase (ALDH) and alcohol dehydrogenase (ADH) are important regulatory genes of alcohol and aldehyde metabolism in animals. ADH transforms all-trans retinoic fat into all-trans retinol in the retinol pathway. All-trans retinol is then converted to all-trans RA through the action of ALDH and the members of the cytochrome 450 superfamily of proteins, CYP2A and CYP3A. The analogous proteins in chicken, CYP26A1, CYP26B1, and CYP26C1, may degrade RA in the retinol pathway and reduce the level of RA in the cell. Of these three enzymes, CYP26B1 has the greatest effect.

**Table 2:** Important regulatory genes in retinol metabolic pathways.

Pathway	The number of genes	P-value	Pathway ID
Retinol metabolism	48	0.51224	ko00830
Primary bile acid biosynthesis	20	0.20024	ko00120
Fatty acid metabolism	27	0.90876	ko00071
Biosynthesis of unsaturated fatty acids	18	0.56182	ko01040
Sphingolipid metabolism	58	0.03153	ko00600
Glycerolipid metabolism	47	0.94096	ko00561
Arachidonic acid metabolism	31	0.68187	ko00590
Synthesis and degradation of ketone bodies	5	0.09865	ko00072
Fatty acid elongation	12	0.84495	ko00062
Fatty acid biosynthesis	5	0.59025	ko00061
Linoleic acid metabolism	15	0.79224	ko00591
alpha-Linolenic acid metabolism	13	0.87191	ko00592
Steroid biosynthesis	8	0.19054	ko00100
Ether lipid metabolism	21	0.99884	ko00565
Glycerophospholipid metabolism	42	0.98306	ko00564
Lipoic acid metabolism	5	0.23253	ko00785
Steroid hormone biosynthesis	38	0.06380	ko00140
Porphyrin and chlorophyll metabolism	24	0.18242	ko00860
Biotin metabolism	2	0.40448	ko00780
Nicotinate and nicotinamide metabolism	19	0.79224	ko00760
Pantothenate and CoA biosynthesis	10	0.95142	ko00770
Riboflavin metabolism	5	0.22910	ko00740
Ubiquinone and other terpenoid-quinone biosynthesis	4	0.43346	ko00130
Folate biosynthesis	6	0.73654	ko00790
Vitamin B6 metabolism	3	0.70346	ko00750
One carbon pool by folate	6	0.97475	ko00670
Thiamine metabolism	1	0.81855	ko00730

**Figure 6:**
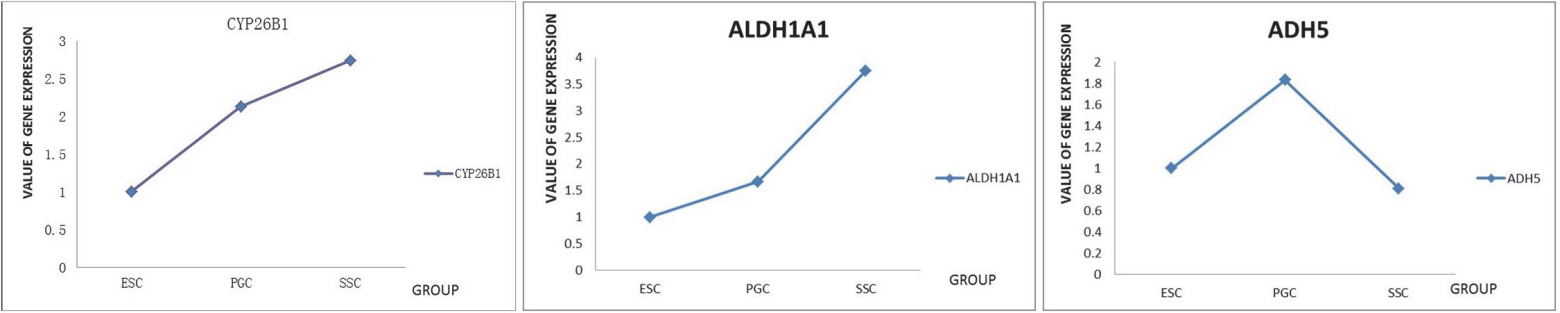
Expression of CYP26b1, ALDH1A1, and ADH5 genes during germ cell differentiation. Analysis of the expression of these genes by qRT-PCR confirmed the expression patterns observed with RNA-seq.

Our results from RNA-seq were consistent with the results from qRT-PCR, confirming the suggestion that these genes are important in regulation of lipid metabolism and retinol metabolism pathways (Fig. 6).

### RA induces ESC differentiation into SSC

**Figure 7:**
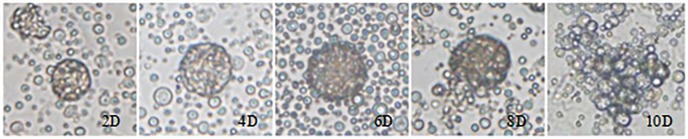
Morphological changes in chicken ESCs during in vitro induction by exogenous RA. *D2*: Note the few, small embryoid bodies. *D4*, *D6*. Embyroid bodies are more numerous and larger than in D2. *D8*. The embryoid bodies are dissociated. *D10*. SSC-like cells are present. Magnification: 400×.

**Figure 8:**
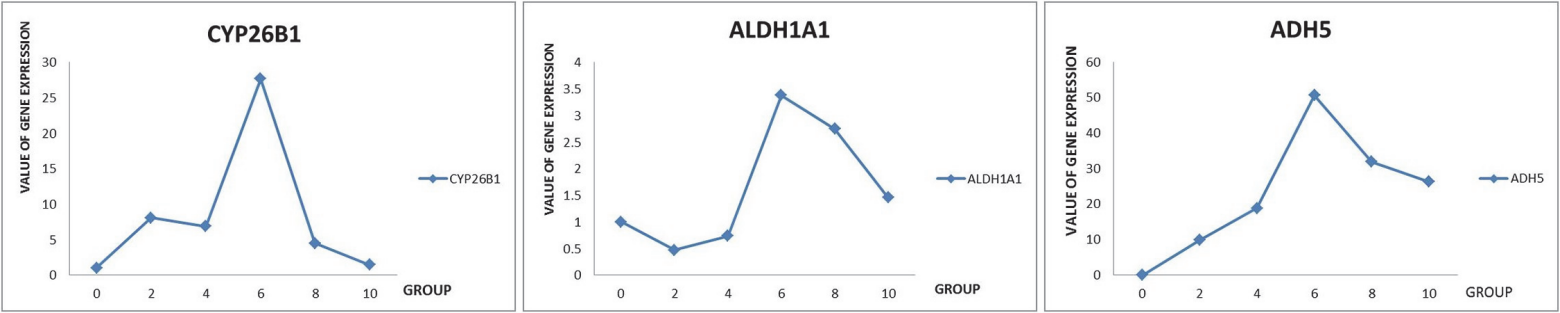
The relative expression value of CYP26B1, ADH1A1, and ADH5 in the induction process. This value was consisent with the results of RNA-seq.

Retinol and its metabolites may regulate the chicken male germ cell differentiation process. To test this role, we applied RA (the retinol pathway activation agent) to ESCs and followed their subsequent development. ESCs were cultured on DMEM rich in glucose and containing 10% FBS and 10 μM RA. The morphology of the ESCs gradually changed, apparently differentiating into SSCs (Fig. 7). The SSC-like cells were labeled with the antibody specific for SSC, confirming that differentiation had occurred. Furthermore, we detected expression of the three key genes of the retinol metabolism pathways through qRT-PCR (Fig. 8).

## Discussion

Chicken was used as a classic model animals of developmental biology, and played an important role in the differentiation research of reproductive stem cell. We adopts the chicken as the materials in this experiment since they have its unique merits: Firstly, it is very convenient to get enough fertilized eggs and help us to get a great deal of ES cells in short time, and not refere to any ethics problems; Secondly, we can conduct operation at any development time in vitro or in vivo since chicken is an ovipara; Thirdly, there is no any report about the RNA-seq sequencing in chicken, especially for the differentiation mechanism of three cell types(ESC, PGC, SSC), so this study have a significant meaning to know about its differentiation mechanism and provide refererene for the subsequent study.

Development and differentiation of the chicken male germ cell is complex. Several metabolic pathways have been implicated in its regulation, including the retinol metabolic pathway [16], Bone Morphogenetic Protein 4 (BMP4) metabolic pathways [17], and the Transforming Growth Factor β (TGFβ) metabolic pathways. In this study, multiple signaling pathways were detected, including lipid, protein, and carbohydrate metabolism that regulate chicken male germ cells differentiation. Twenty-seven metabolic signaling pathways were detected, mostly involving steroid hormone biosynthesis, synthesis of primary bile acids, retinol metabolism, fatty acid metabolism, and triglyceride metabolism.

The current research focused on the importance of lipid metabolism in cell differentiation, a proposal that is supported by considerable evidence. In mice male germ cells, for example, the expression of proteins in the Fatty Acid Binding proteins (FABPs) family and the expression of Diacylglycerol Acyltransferase (DGAT) varied during differentiation and development. Specifically, FABP9, FABP12, and DGAT were up-regulated and highly expressed throughout the entire differentiation process of sperm cells [18]. Also, Fujisawa et al. [19] found that adding human milk to the medium of 3T3-L1 cells induced their differentiation. Many other studies support the importance of lipid metabolism in the in vitro induction of differentiation of ESCs into male germ cells. Pan Shaohui [20] induced differentiation of mouse ESCs with the exogenous steroids estradiol and follicle-stimulating hormone, which was accompanied by increased expression of Intergerin, Dazl, Scp3, and Vasa, genes specific to germ cells, as revealed by immune-fluorescence seven days after induction. Likewise, another group working with pig skin germ cells supplemented the culture medium with follicular fluid and FBS, inducing differentiation and expression of Oct4, Vasa, and Dazl. Moreover, estrogen and progesterone were detected in the cluster after differentiation [21]. In vitro differentiation of human embryonic stem cells (hESC) may be stimulated by steroid hormones [22]. Finally, several studies have shown that the final product of retinol metabolism pathways, RA, can induce differentiation of ESCs to the male germ cell in vitro and produce SSC-like cells. Although these observations implicate lipid metabolism in the regulation of stem cell differentiation, there has not been a systemic study on the regulatory mechanism or the related genes.

To this end, we have screened the key signaling pathways and genes that participate in the regulation of the male germ cell differentiation process by DNA microarray, RNA-seq, verification with qRT-PCR, and an in vitro induction experiment. The classic models for studying differentiation and development in the animal germ cell have been mice and fruit flies, but chicken embryos offer certain advantages over these. They are easily accessed in large numbers and researchers are beginning to recognize these benefits.

Our research group is committed to using this system in researching the differentiation of the domestic chicken male germ cell. Previous studies suggested that ADH, ALDH, and the cytochrome P450 family members of the RA metabolism pathway are involved in regulation of the male germ cell differentiation in poultry. The CYP1 family members catalyze retinol into retinal, which is further metabolized to RA. Several studies have shown that CYP1 family members were down-expressed when the receptor of CYP1 was knocked out, accompanied by reduced levels of RA [23]. From pathway analysis of the differentiation process of male chicken germ cells, the main regulator of retinol metabolism is CYP1A4, but CYP26 is the key enzyme in the regulation of RA level in vivo, and acts by converting RA back to retinol. The CYP26 family contains CYP26A1, CYP26B1, and CYP26C1, which are all relatively evolutionarily conserved. Cultivated embryos of the African clawed frog that had been previously injected with CYP26A1 mRNA presented with symptoms of RA deficiency, supporting the involvement of CYP26A1 in the regulation of RA levels and the normal development of embryos in vivo [24]. Koubova achieved overexpression of CYP26B1 in COS-1 cells, and observed that RA was converted to retinol, which suggested that CYP26B1 participated in RA metabolism. The cultured embryos also had the same symptoms as embryos over-expressing CYP26C1 or embryos that had been treated with a retinaldehyde dehydrogenase inhibitor. These observations suggest that CYP26C1 is involved in RA metabolism [25]. CYP26 can be induced by RA, which is supported by our results in the induction experiment reported here. Based on our results and those of others, we conclude that CYP26 family members are the main CYP450 enzyme in RA metabolism in vivo. In this study, we demonstrated the expression of CYP450 family members with RNA-seq, and then verified our finding with qRT-PCR. We also discovered that expression of the CYP450 family members was induced by exogenous RA in vitro. We suggest that this family plays an important role in retinol metabolism, which has an important effect in the differentiation process of male germ cells.

Recent studies have shown that RA is important in the sex differentiation process of domestic chickens. Ovarian levels of RA apparently reach a certain threshold in chicken PGCs that are at a developmental stage of 15.51 E. Then, RA induces the expression of *Stra8* and a series of genes, and induces the germ cells to proceed to meiosis. In the testis, however, expression of CYP26b1 inhibits the over-expression of RA and delays meiosis in the germ cells [26, 27].

RA at 10 μM was shown to induce mice ESC to differentiate into male germ cells and chicken ESC to differentiate into SSC-like cells; both of these studies were in vitro. Based on these studies and the fact that retinol is the final product of the retinol pathways, we used RA to explore expression of the major genes in retinol metabolic pathways during the differentiation process in vitro. We found that the key genes of the retinol pathway were affected by RA added to the culture medium, and it stimulated differentiation of the germ cells. RA can stimulate up-regulation of CYP26B1, but then the enzyme will cause it to be degraded. Based on others’ work, RA may have mediated *stra8* expression, starting meiosis, but then its action could have been reversed by the action of CYP26B1. The level of RA may have increased to mediate *Stra8* expression and begin differentiation, but the levels would not have stayed high.

Our experimental results show that key genes of the retinol metabolic pathways in vitro were induced by RA. This suggests that these genes play a major regulatory role in the retinol metabolic pathway. The success of in vitro induction also verified the result of RNA-seq and verifies the importance of the retinol metabolic pathway in the development and differentiation of chicken male germ cells. We conclude that 10 μM RA and Sertoli cells as the feeding layer may provide optimal induction conditions.

In summary, we studied the role of lipid metabolism in differentiation of male germ cells from chicken embryos. We focused on metabolic pathways of retinol and used RNA-seq, qRT-PCR, and gene chip technologies combined with induction of differentiation by RA, to analyze these pathways in detail. Results indicated that RA could induce ESC to differentiation into male germ cells in vitro. We hope that this study lays a solid foundation for future work.

## References

[1] Lu JX, Liu XZ, Zang RX, Chen FF, Yang GG, et al (2006). Studies on expression orders of lipidmetabolism-related genes during differentiation of rat adipocytes. Veterinary Science in China, 36: 306–310

[2] Kee K, Angeles V, Flores M, Nguyen H, Pera R (2009). Human DAZL, DAZ and BOULE genes modulate primordial germ-cell and haploid gamete formation. Nature, 462: 222–225 10.1038/nature08562 19865085 PMC3133736

[3] Aflatoonian B, Moore H (2005). Human primordial germ cells and embryonic germ cells,And their use in cell therapy. Current opinion in Biotechnology, 16: 530–535. 16154336 10.1016/j.copbio.2005.08.008

[4] Hübner K, Fuhrmann G, Christenso LK, Kehler J, Reinbold R, et al (2003). Derivation of oocytes from mouse embryonic stem cells. Science, 300: 1251–1256. 12730498 10.1126/science.1083452

[5] Costa V, Angelini C, De FI, Ciccodicola A (2010). Uncovering the complexity of transcriptomes with RNA-Seq. Journal of Biomedicine and Biotechnology. 10.1155/2010/853916 PMC2896904 20625424

[6] Wang Z, Gerstein M, Snyder M (2009). RNA-Seq: a revolution-ary tool for transcriptomics. Nat Rev Genet, 10: 57–63. 10.1038/nrg2484 19015660 PMC2949280

[7] Cloonan N, Forrest AR, Kolle G, Gardiner BBR, Faulkner GJ, et al(2008). Stem cell transcriptome profiling via massive-scale mRNA sequencing. Nature methods, 5: 613–619. 10.1038/nmeth.1223 18516046

[8] Mortazavi A, Williams BA, McCue K, Schaeffer L, Wold B, et al(2008). Mapping and quantifying mammalian transcriptomes by RNA-Seq. Nature methods, 5: 621–628. 10.1038/nmeth.1226 18516045 PMC13303166

[9] Nagalakshmi U, Wang Z, Waern K, Shou C, Raha D, et al(2008). The transcriptional landscape of the yeast genome defined by RNA sequencing. Science, 320: 1344–1349. 10.1126/science.1158441 18451266 PMC2951732

[10] Gan Q, Chepelev I, Wei G, Tarayrah L, Cui K, et al (2010). Dynamic regulation of alternative splicing and chromatin structure in Drosophila gonads revealed by RNA-seq. Cell research, 20: 763–783. 10.1038/cr.2010.64 20440302 PMC2919574

[11] Li BC, Cheng GH, Zhao DW, Wang KH, Qian JF, et al(2002). Relationship between migration and gonad development in the early chicken embryo.Journal of yangzhou university, 23: 18–26.

[12] Wu XS, He XH, Dai JM, Tian ZQ, Yang HY, et al (2008). Isolation and Cultivation of Chicken Embryonic Stem Cells and Production of Single-cell Clone. ChinaPoultry, 30: 30–34.

[13] Tang XY, Zeng WD, Mi YL, Liu HY, Zhang CQ (2006). Isolation, Culture and Characterization of Chicken Primordial Germ Cells. Journal of agricultural biotechnology,14: 174–177.

[14] Sun SY, Li BC, Wei CX, Qin J, Wu H, et al (2008). Culture of chicken spermatagonial stem cells in vitro. Chinese Journal of Veterinary, 28: 102–105.

[15] Sun M (2012). Studyon Male Germ Cell Derived from ChickenEmbryonicStem Cell and the Generation of Transgenic Chicken Yangzhou university.

[16] Kerkis A, Fonseca SA, Serafim RC, Lavagnolli TMC, Abdelmassih S, et al (2007). In vitro differentiation of male mouse embryonic stem cells into both presumptive sperm cells and oocytes.Cloning and stem cells, 9: 535–548. 18154514 10.1089/clo.2007.0031

[17] Toyooka Y, Tsunekawa N, Akasu R, Noce T (2003). Embryonic stem cells can form germ cells in vitro. Proceedings of the National Academy of Sciences, 100: 11457–11462. 14504407 10.1073/pnas.1932826100 PMC208779

[18] Oresti GM, García LJ, Aveldaño MI, Mazo JD (2013). Cell-type-specific regulation of genes involved in testicular lipid metabolism: fatty acid-binding proteins, diacylglycerol acyltransferases, and perilipin 2. Reproduction, 146: 471–480. 10.1530/REP-13-0199 23962454

[19] Fujisawa Y, Yamaguchi R, Nagata E, Satake E, Sano S, et al(2013). The lipid fraction of human milk initiates adipocyte differentiation in 3T3-L1 cells. Early human development, 89: 713–719. 10.1016/j.earlhumdev.2013.05.002 23759379

[20] Pan SH (2010). Study on mouse embryonic stem cells differention into male germ cells Northwest Agriculture & Forestry University

[21] Dyce P, Wen L, Li J (2006). In vitro germline potential of stem cells derived from fetal porcine skin. Nature cell biology, 8: 384–390. 16565707 10.1038/ncb1388

[22] Dong YL, Liu JY, Wang KH, Lu XM (2011). Major research advance on derivation of germ cells from embryonic stem cells. CHINESE JOURNAL OF FAMILY PLANNING, 19: 247–249.

[23] Andreola F, Fernandez-Salguero PM, Chiantore MV, Petkovich MP, Gonzalez FJ, et al (1997). Aryl hydrocarbon receptor knockout mice (AHR−/−) exhibit liver retinoid accumulation and reduced retinoic acid metabolism. Cancer research, 57: 2835–2838. 9230184

[24] Trofimova-Griffin ME, Brzezinski MR, Juchau MR (2000). Patterns of CYP26 expression in human prenatal cephalic and hepatic tissues indicate an important role during early brain development. Developmental Brain Research, 120: 7–16. 10727725 10.1016/s0165-3806(99)00185-6

[25] Wang P, Liu XD (2011). Cytochrome P450s and the metabolism of vitamin A. CHINESE JOURNAL OF CLINICAL PHARMACOLOGY AND THERAPEUTICS, 16: 474–480.

[26] McLaren A (2003). Primordial germ cells in the mouse. Developmental biology, 262: 1–15. 14512014 10.1016/s0012-1606(03)00214-8

[27] Koubova J, Menke DB, Zhou Q, Capel B, Griswold MD, et al (2006). Retinoic acid regulates sex-specific timing of meiotic initiation in mice. Proceedings of the National Academy of Scien, 103: 2474–2479 16461896 10.1073/pnas.0510813103 PMC1413806

